# scEccDNAdb: an integrated single-cell eccDNA resource for human and mouse

**DOI:** 10.1093/database/baae126

**Published:** 2024-12-18

**Authors:** Wenqing Wang, Xinyu Zhao, Tianyu Ma, Tengwei Zhong, Junnuo Zheng, Zhiyun Guo

**Affiliations:** School of Life Sciences and Engineering, Southwest Jiaotong University, No.111, North 1st Section of Second Ring Road, Chengdu, Sichuan 610031, China; School of Life Sciences and Engineering, Southwest Jiaotong University, No.111, North 1st Section of Second Ring Road, Chengdu, Sichuan 610031, China; School of Life Sciences and Engineering, Southwest Jiaotong University, No.111, North 1st Section of Second Ring Road, Chengdu, Sichuan 610031, China; School of Life Sciences and Engineering, Southwest Jiaotong University, No.111, North 1st Section of Second Ring Road, Chengdu, Sichuan 610031, China; School of Life Sciences and Engineering, Southwest Jiaotong University, No.111, North 1st Section of Second Ring Road, Chengdu, Sichuan 610031, China; School of Life Sciences and Engineering, Southwest Jiaotong University, No.111, North 1st Section of Second Ring Road, Chengdu, Sichuan 610031, China

## Abstract

Extrachromosomal circular DNA (eccDNA), an extrachromosomal circular structured DNA, is extensively found in eukaryotes. Investigating eccDNA at the single-cell level is crucial for understanding cellular heterogeneity, evolution, development, and specific cellular functions. However, high-throughput identification methods for single-cell eccDNA are complex, and the lack of mature, widely applicable technologies has resulted in limited resources. To address this gap, we built scEccDNAdb, a database based on single-cell whole-genome sequencing data. It contains 3 195 464 single-cell eccDNA entries from human and mouse samples, with annotations including oncogenes, typical enhancers, super-enhancers, CCCTC-binding factor-binding sites, single nucleotide polymorphisms, chromatin accessibility, expression quantitative trait loci, transcription factor binding sites, motifs, and structural variants. Additionally, it provides nine online analysis and visualization tools, which enable the creation of publication-quality figures through user-uploaded files. Overall, scEccDNAdb is a comprehensive database for analyzing single-cell eccDNA data across diverse cell types, tissues, and species.

**Database URL**: https://lcbb.swjtu.edu.cn/scEccDNAdb/

## Introduction

Extrachromosomal circular DNA (eccDNA), characterized by its circular structure outside the chromosomes, is widely present in eukaryotes and plays crucial roles in tumorigenesis and immune response, which can function as a biomarker [1, 2]. However, presently eccDNA researches predominantly focus on the bulk level, potentially equalizing the cellular diversity, which may cause the information loss in single-cell eccDNA. Additionally, low-copy eccDNAs or those in limited cells may remain undetected by conventional bulk methods [3]. In contrast, single-cell approaches address these limitations, providing deeper insights into ecDNA’s heterogeneity, which results in heterogeneous transcriptional regulation [4, 5], as well as dynamic cell cycle changes, cancer evolution [6, 7], cellular functions, and regulation at the single-cell level [8–10].

Currently, single-cell eccDNA research mainly relies on sequencing technologies like SMOOTH-seq [9], scEC&T-seq [7], scCircle-seq [6], and scGTP-seq [11]. However, challenges such as enrichment difficulty due to the circular nature of eccDNA and cost constraints have interfered with the development of standardized high-throughput technologies for single-cell eccDNA identification. Currently, eccDNA identification methods based on short-read whole-genome sequencing (WGS) data have been widely used [12]. However, considering the extensive emergence of single-cell WGS data, these data are dispersed across various literature and datasets. There is an urgent need to establish a dedicated repository for single-cell eccDNA research to leverage these valuable data and advance this field.

**Figure 1.**
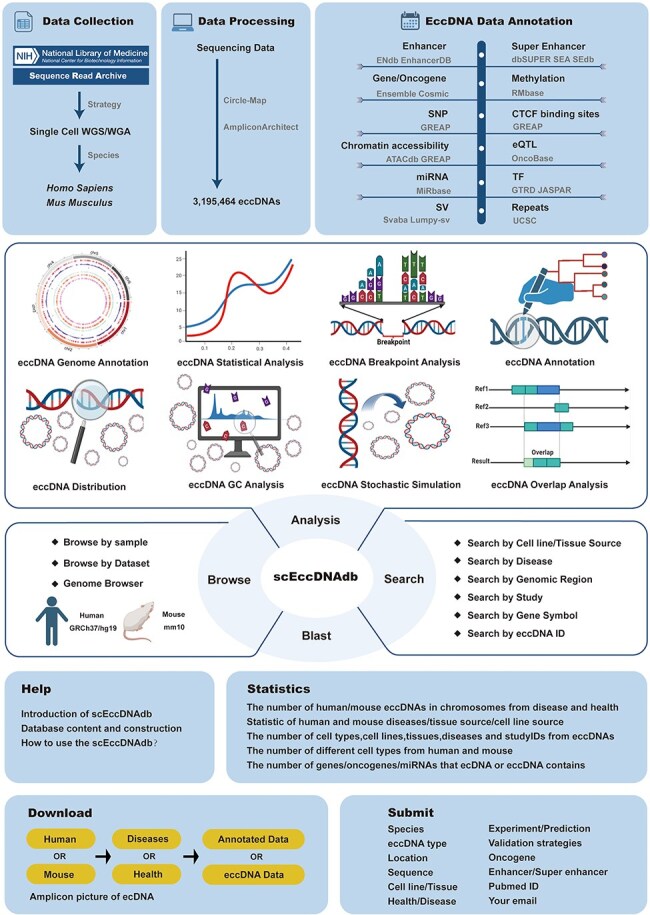
A schematic workflow of scEccDNAdb.

To date, several eccDNA databases have been established, including CircleBase [13], eccDB [14], eccDNA Atlas [15], eccDNAdb [16], TeCD [17], and EccBase [18]. While valuable, these are all bulk eccDNA databases and no large-scale single-cell eccDNA resource exists. Here, we identified eccDNAs from 3538 single-cell WGS datasets and developed the first comprehensive single-cell eccDNA database as far as we know, named “scEccDNAdb.” It features a user-friendly web interface for exploring detailed eccDNA information and annotations. Additionally, it offers tools such as search, blast, browse, and analysis for in-depth data exploration. Figure 1 illustrates the database construction process.

## Materials and methods

### Data collection

In the NCBI-SRA database, single-cell WGS data for human and mouse were collected using three keywords: (i) ((((“single cell“[All Fields] OR “single-cell“[All Fields] OR “single cells“[All Fields] OR “single-cells“[All Fields]) AND WGA[Strategy]))); (ii) ((((“single cell”[All Fields] OR “single-cell”[All Fields] OR “single cells”[All Fields] OR “single-cells”[All Fields]) AND WGS[Strategy]))); and (iii) “scWGS.” After removing duplicates and retaining paired-end data, manual corrections were applied. A total of 2163 disease and 1027 healthy human samples, along with 79 disease and 269 healthy mouse samples, were obtained.

### Single-cell eccDNA identification

Quality control of WGS data was performed using Fastp (v0.23.2) with default settings. BWA (v0.7.17) software was used to align reads to reference genomes (human hg19, mouse mm10). Sorting and duplicate read filtering were performed using SAMtools (v1.7) and Sambamba (v0.8.2) software. EccDNA amplicons were identified using AmpliconArchitect (v1.0.0) [19] based on the criteria of genome fragments >50 kb and copy numbers (CNs) ≥4.5. AmpliconClassifier was used to classify predicted amplicons. Circle-Map (v1.1.4) [20] was used for eccDNA identification, retaining results with “split read ≥1” (users also can apply various quality filters based on the split read criteria provided by scEccDNAdb) and “size <1 kb” according to the previous study [21].

### Annotation and visualization

All eccDNA sequences in scEccDNAdb were converted to the default hg19 and mm10 genome versions using the UCSC LiftOver tool. Regulatory elements located entirely within the eccDNA region were annotated as eccDNA regulatory elements. Detailed statistics are provided in Table 1. Circular plots of eccDNA were created using Circos. The UCSC browser was utilized for visualizing annotation of both the default existing and user-defined eccDNA regions.

**Table 1.** Annotation data of eccDNAs in scEccDNAdb

Annotation type	Numbers	Source
Gene	2 001 501	Ensembl
Oncogene	77 345	COSMIC
MiRNA	2705	miRbase
Methylation sites (m1A, m6A, and m5C)	242 063	RMBase
Repeats	3 630 814	UCSC
Typical enhancers	116 941	ENdb&EnhancerDB
Super enhancers	336 763	dbSUPER and SEA&SEdb
CTCF-binding sites	499 254	GREAP
SNP	211 948	GREAP
Chromatin accessibility	132 567	ATACdb& GREAP
eQTL	341 316	OncoBase
TF-binding sites	11 499 252	GTRD&JASPER

### Blast, structural variations, and motif enrichment analysis

BLAST was constructed using the ViroBLAST [22] online tool. MakeBlastdb was employed to create nucleic acid sequence libraries for human and mouse in scEccDNAdb. For human samples, sequences aligned to the reference genome were sorted and indexed using SAMtools, and then structural variants (SVs) were detected in eccDNA regions using SvABA (v1.1.0). For mouse samples, discordant paired-end and split-read alignments were extracted from sequences aligned to the reference genome, and SV detection was performed using lumpy-sv (v0.2.13). Then, SQL was used to match the SV results to each mouse eccDNA. Motif enrichment analysis was conducted using Homer (v4.11) with the findMotifsGenome.pl command to detect motifs within each single-cell eccDNA region of 200 bp.

### Analysis module development

The single-cell eccDNA analysis comprises eight modules, each developed and executed using R Shiny, with the system() function invoking Linux commands during analysis: (I) eccDNA genome annotation: users input customized chromosome positions for annotation using a PHP function; (II) statistical analysis: statistical graphs were generated using the R package ggplot2; (III) breakpoint analysis: the R package ggseqlogo was used to analyze and plot sequence distribution patterns within ±10 bp around eccDNA breakpoints; (IV) annotation analysis: the HOMER (v4.11) script annotatePeaks.pl (hg19, with the -annStats parameter) was used to capture element types within specified eccDNA regions, with statistical graphs generated using the R package ggplot2; (V) distribution analysis: the R package RIdeogram was used to plot chromosome distribution of single-cell eccDNA; (VI) GC analysis: the computeMatrix scale-regions function in deeptools (v3.5.5) calculated signal density in specified regions, while the plotProfile function (with default parameters) generated signal plots of GC content in eccDNA regions; (VII) stochastic simulation: the shuffle function in bedtools (v2.31.0) (with parameters -i, -excl, and -g ) was used to generate simulated regions similar to eccDNA regions submitted by the user; and (VIII) overlap analysis: the intersect function in bedtools (v2.31.0) (with parameters -a, -b, -wa, -wb, and -f) captured eccDNA regions in scEccDNAdb that overlap with user-submitted eccDNA regions. The analysis outputs publication-quality images at 300 dpi.

### System design and implementation

The scEccDNAdb website operates on a Nginx Web server (https://nginx.org/). The database was developed using MySQL (5.7.27) (http://www.mysql.com), with PHP (7.2.30) (http://www.php.net) for server-side scripting. The web interface was built using Bootstrap (v3.3.7) (https://v3.bootcss.com), JQuery (v2.1.1) (http://jquery.com), and ECharts (http://echarts.baidu.com) for graphical visualization.

## Results

**Figure 2.**
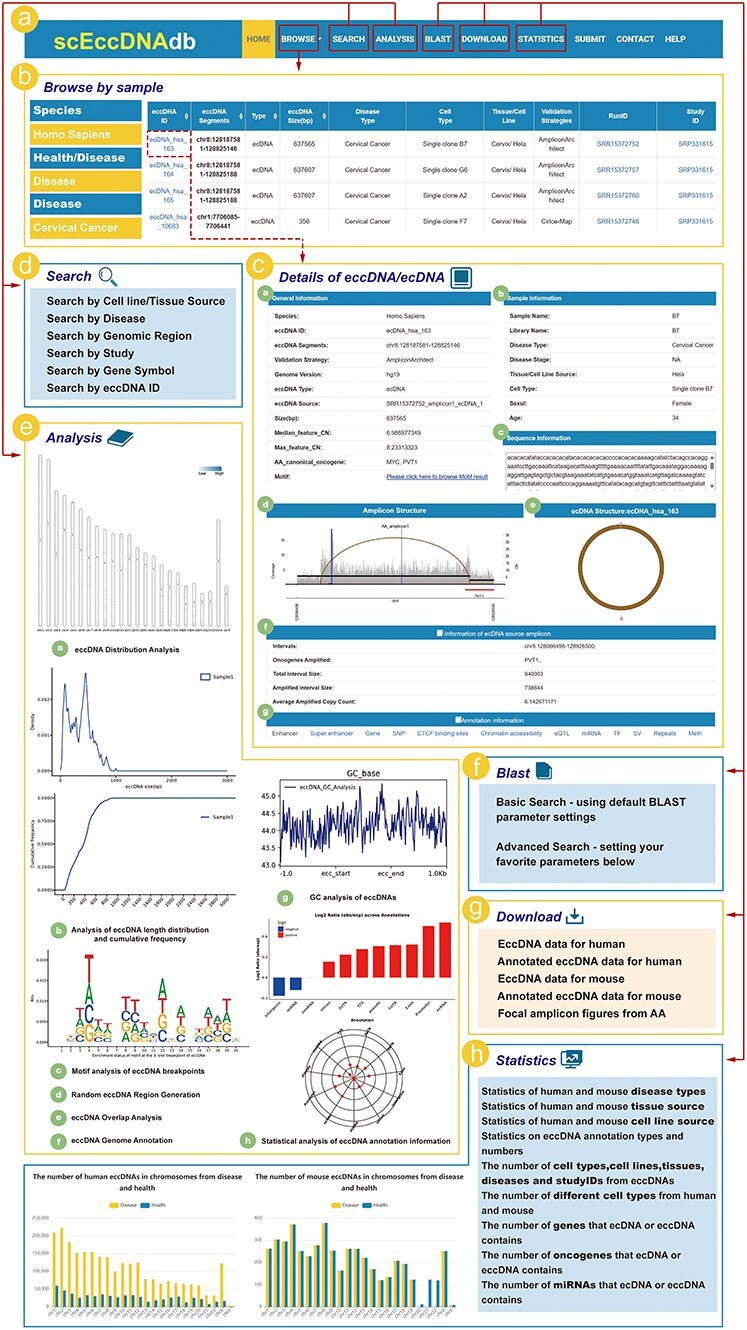
The web interface and usage of scEccDNAdb. (a) The navigation bar of scEccDNAdb. (b) Users can browse eccDNA data by sample and datasets. (c) “Details of eccDNA/ecDNA” includes (i) General information, (ii) Sample information, (iii) Sequence information, (iv) Amplicon structure, (v) Circular schematic of eccDNA, (vi) Information of ecDNA source amplicon, and (vii) Annotation information. (d) Users can query eccDNA data in six ways. (e) The eight analysis modules of scEccDNAdb. (f) Blast tool. (g) Data download page. (h) Statistics of scEccDNAdb.

ScEccDNAdb consists of seven main sections: (i) BROWSE, (ii) SEARCH, (iii) ANALYSIS, (iv) BLAST, (v) DOWNLOAD, (vi) STATISTICS, and (vii) SUBMIT (Fig. 2a). The detailed web interface and usage are as follows.

### The browse and search module

The “Browse” page has three menus: “Browse by sample,” “Browse by datasets,” and “Genome browser” (Fig. 2b). “Browse by sample” enables users to explore eccDNA by species, disease type (health/disease), and cell type (tissue source/cell line source). “Browse by datasets” allows users to browse eccDNA by species, disease type, and study ID. Users can click on an eccDNA ID to access detailed information about that eccDNA (Fig. 2c). The “Genome browser” is a visualization tool based on the UCSC genome browser, enabling users to visualize eccDNA genomic regions. Users can select a specific eccDNA from scEccDNAdb or enter a custom region to view histone modification signals, mutations, sequence conservation, and other relevant information.

In the SEARCH module, users can query eccDNA data using six approaches: (i) “Search by tissue/cell line source” for searching eccDNAs through three steps, including “species,” “disease/health,” and “cell line”; (ii) “Search by Disease” for searching eccDNAs based on different disease types in humans or mouse; (iii) “Search by region” for searching chromosome locations of interest by users of all eccDNAs we collected; (iv) “Search by study” for searching eccDNAs from the studies used for eccDNA identification; (v) “Search by gene symbol” for searching the gene on eccDNA by name; and (vi) “Search by eccDNA ID” (Fig. 2d).

### EccDNA single-cell analysis module

The ANALYSIS module of scEccDNAdb includes the following eight parts (Fig. 2e): (i) “eccDNA Distribution Analysis” generates publication-quality figures for eccDNA chromosomal distribution in a single or paired sample by uploading a customized file. (ii) “eccDNA Statistical Analysis” generates publication-quality figures for length distribution and cumulative frequency of eccDNA in a single or paired sample by uploading a customized bed file. (iii) “eccDNA Breakpoint Analysis” generates publication-quality sequence logos (motif enrichment) in the ±10 bp region around the eccDNA breakpoints by uploading a customized file. (iv) “eccDNA Stochastic Simulation” generates random eccDNA regions of the same size as the user uploads a customized file of eccDNA regions. (v) “eccDNA Overlap Analysis” performs overlap analysis between customized eccDNA regions and eccDNA regions of scEccDNAdb. (vi) “eccDNA Genome Annotation” performs annotation analysis on eccDNA regions or custom chromatin regions, including oncogenes/lncRNAs, typical enhancers, super-enhancers, CCCTC-binding factor (CTCF)-binding sites, single nucleotide polymorphisma (SNPs), chromatin accessibility, and expression quantitative trait loci (eQTLs). Furthermore, we provide external links for eccDNA details, including GEPIA [23], GeneCard [24], and String databases [25]. (vii) “eccDNA GC Analysis” generates GC enrichment figures of eccDNAs from a customized file. (viii) “eccDNA Annotation Analysis” observes the enrichment and quantity distribution of annotated elements of single-cell eccDNA for a specific period.

### BLAST

The BLAST tool helps users identify local similarities between query and eccDNA sequences. Users can upload or paste DNA sequences in FASTA format and select datasets for the BLAST search (Fig. 2f).

### Download, submit, and statistics

Users can download eccDNA data (Fig. 2g) and share research data (e.g. location and sequence) through the submission interface. The statistics page classifies and counts eccDNA data by chromosome, tissue, cell line, disease, etc. (Fig. 2h).

### A case study

Taking single-cell eccDNA of cervical cancer as an example, the detailed application of scEccDNAdb is as follows: in the “browse by sample” interface, users can access a list of data containing all eccDNA samples from single cells of cervical cancer by clicking on the “Cervical Cancer” label. In the search interface, using the “Search eccDNA by Disease” strategy, users can select “Homo sapiens” as the species and “Cervical Cancer” as the disease type in Steps 1 and 2, respectively, to retrieve data on 4626 eccDNAs from cervical cancer samples. The search results page provides information including eccDNA ID, eccDNA segments, eccDNA size (bp), disease type, cell type, tissue/cell type, validation strategies, run ID, and study ID for each eccDNA. Furthermore, users can refine the results list using the “Search” box located at the top left corner.

Taking “ecDNA_hsa_163” from the results list as an example, users can click on the eccDNA ID to gain detailed insights into the specific eccDNA. The “General information” in the “Detail” interface reveals that this eccDNA spans the region chr8:128187581-128825146, sourced from the single-cell clone sample “B7” of the “Hela” cell line, with a size of 637 565 bp. The “Sequence information” presents the nucleotide arrangement of this eccDNA region. The “Median_feature_CN” is “6.988977349,” and the “Max_feature_CN” is “8.23313323.” Notably, the annotation identifies the oncogenes “MYC” and “PVT1” within ecDNA_hsa_163, both known to play crucial roles in tumorigenesis. Previous studies have shown that the “PVT1” promoter is ectopically fused with “MYC” and duplicated in ecDNA, resulting in potent “MYC” expression mediated through enhancer hijacking [26]. The ecDNA structure diagram illustrates the circular structure of ecDNA_hsa_163. The “Amplicon structure” delineates the amplification structure of the eccDNA source, while the “Information of ecDNA source amplicon” section illustrates pertinent details such as the interval located at chr8:128086498-128926500, amplified oncogenes including “PVT1”, and total interval size of 84 0003 bp. Finally, the “annotation information” encompasses annotations on the ecDNA_hsa_163, including 55 enhancers, 40 super-enhancers, 9 genes (including the oncogenes “MYC” and “PVT1”), 3534 CTCF-binding sites, 5117 chromatin accessibility regions, 31 eQTLs, 2 miRNAs, 494 Transcription factor (TFs), 1441 SVs, 1436 repeats, and 80 methylation regions.

In conclusion, the data extracted from this database are of paramount importance for advancing research into the potential functions and regulatory roles of eccDNAs in diseases.

## Discussion and conclusion

We collected publicly available single-cell WGS data to identify eccDNA and developed the scEccDNAdb database. scEccDNAdb is a unique repository for single-cell eccDNA, offering extensive datasets from human and mouse samples, including metadata, genomic sequences, and detailed annotations. Moreover, scEccDNAdb provides online tools for analysis and visualization, helping users explore eccDNA biogenesis and regulatory mechanisms. However, challenges remain in eccDNA identification and analysis. For example, single-cell WGS data are often low-coverage WGS, effectively affecting eccDNA identification. Additionally, the lack of omics data linked to cell types and clinical information also complicates comprehensive analysis.

As single-cell technologies advance, more omics data will emerge, enhancing our understanding of eccDNA. We will continuously update scEccDNAdb with the latest single-cell datasets, including scWGS and potentially scATAC-seq, and plan to integrate additional eccDNA analysis tools, such as eccDNA-pipe [27], CReSIL [28], Circlehunter [29], Deepcircle [30], and ATACAmp [31]. We also aim to develop new analysis features to improve database interactivity, offering users a better experience. By consistently using and refining scEccDNAdb, our goal is to deepen our understanding of eccDNA single-cell heterogeneity, especially its roles in intratumoral heterogeneity and cancer evolution.

## Data Availability

ScEccDNAdb is freely available online at https://lcbb.swjtu.edu.cn/scEccDNAdb/.
